# A Review of Data Quality Assessment Methods for Public Health Information Systems

**DOI:** 10.3390/ijerph110505170

**Published:** 2014-05-14

**Authors:** Hong Chen, David Hailey, Ning Wang, Ping Yu

**Affiliations:** 1 School of Information Systems and Technology, Faculty of Engineering and Information Sciences, University of Wollongong, Wollongong, NSW, 2522, Australia; E-Mails: hc978@uowmail.edu.au (H.C.); dhailey@ozmail.com.au (D.H.); 2 Jiangxi Provincial Center for Disease Control and Prevention, Nanchang 330029, China; 3 National Center for AIDS/STD Control and Prevention, Chinese Center for Disease Control and Prevention, Beijing 102206, China; E-Mail: wangnbj@163.com

**Keywords:** data quality, information quality, data use, data collection process, evaluation, assessment, public health, population health, information systems

## Abstract

High quality data and effective data quality assessment are required for accurately evaluating the impact of public health interventions and measuring public health outcomes. Data, data use, and data collection process, as the three dimensions of data quality, all need to be assessed for overall data quality assessment. We reviewed current data quality assessment methods. The relevant study was identified in major databases and well-known institutional websites. We found the dimension of data was most frequently assessed. Completeness, accuracy, and timeliness were the three most-used attributes among a total of 49 attributes of data quality. The major quantitative assessment methods were descriptive surveys and data audits, whereas the common qualitative assessment methods were interview and documentation review. The limitations of the reviewed studies included inattentiveness to data use and data collection process, inconsistency in the definition of attributes of data quality, failure to address data users’ concerns and a lack of systematic procedures in data quality assessment. This review study is limited by the coverage of the databases and the breadth of public health information systems. Further research could develop consistent data quality definitions and attributes. More research efforts should be given to assess the quality of data use and the quality of data collection process.

## 1. Introduction

Public health is “the science and art of preventing disease, prolonging life, and promoting physical health and efficiency through organized community efforts” [1]. The ultimate goal of public health is to improve health at the population level, and this is achieved through the collective mechanisms and actions of public health authorities within the government context [1,2]. Three functions of public health agencies have been defined: assessment of health status and health needs, policy development to serve the public interest, and assurance that necessary services are provided [2,3]. Since data, information and knowledge underpin these three functions, public health is inherently a data-intensive domain [3,4]. High quality data are the prerequisite for better information, better decision-making and better population health [5].

Public health data represent and reflect the health and wellbeing of the population, the determinants of health, public health interventions and system resources [6]. The data on health and wellbeing comprise measures of mortality, ill health, and disability. The levels and distribution of the determinants of health are measured in terms of biomedical, behavioral, socioeconomic and environmental risk factors. Data on public health interventions include prevention and health promotion activities, while those on system resources encompass material, funding, workforce, and other information [6].

Public health data are used to monitor trends in the health and wellbeing of the community and of health determinants. Also, they are used to assess the risks of adverse health effects associated with certain determinants, and the positive effects associated with protective factors. The data inform the development of public health policy and the establishment of priorities for investment in interventions aimed at modifying health determinants. They are also used to monitor and evaluate the implementation, cost and outcomes of public health interventions, and to implement surveillance of emerging health issues [6]. 

Thus, public health data can help public health agencies to make appropriate decisions, take effective and efficient action, and evaluate the outcomes [7,8]. For example, health indicators set up the goals for the relevant government-funded public health agencies [5]. Well-known health indicators are the Millennium Development Goals (MDGs) 2015 for the United Nations member states [9]; the European Core Health Indicators for member countries of the European Union [10]; “Healthy People” in the United States, which set up 10-year national objectives for improving the health of US citizens [11]; “Australia: The Healthiest Country by 2020” that battles lifestyle risk factors for chronic disease [12]; and “Healthy China 2020”, an important health strategy to improve the public’s health in China [13].

Public health data are generated from public health practice, with data sources being population-based and institution-based [5,6]. Population-based data are collected through censuses, civil registrations, and population surveys. Institution-based data are obtained from individual health records and administrative records of health institutions [5]. The data stored in public health information systems (PHIS) must first undergo collection, storage, processing, and compilation. The procured data can then be retrieved, analyzed, and disseminated. Finally, the data will be used for decision-making to guide public health practice [5]. Therefore, the data flows in a public health practice lifecycle consist of three phases: data, data collection process and use of data. 

PHIS, whether paper-based or electronic, are the repositories of public health data. The systematic application of information and communication technologies (ICTs) to public health has seen the proliferation of computerized PHIS around the world [14,15,16]. These distributed systems collect coordinated, timely, and useful multi-source data, such as those collected by nation-wide PHIS from health and other sectors [17]. These systems are usually population-based, and recognized by government-owned public health agencies [18].

The computerized PHIS are developed with broad objectives, such as to provide alerts and early warning, support public health management, stimulate research, and to assist health status and trend analyses [19]. Significant advantages of PHIS are their capability of electronic data collection, as well as the transmission and interchange of data, to promote public health agencies’ timely access to information [15,20]. The automated mechanisms of numeric checks and alerts can improve validity and reliability of the data collected. These functions contribute to data management, thereby leading to the improvement in data quality [21,22]. 

Negative effects of poor data quality, however, have often been reported. For example, Australian researchers reported coding errors due to poor quality documentations in the clinical information systems. These errors had consequently led to inaccurate hospital performance measurement, inappropriate allocation of health funding, and failure in public health surveillance [23]. 

The establishment of information systems driven by the needs of single-disease programs may cause excessive data demand and fragmented PHIS systems, which undermine data quality [5,24]. Studies in China, the United Kingdom and Pakistan reported data users’ lack of trust in the quality of AIDS, cancer, and health management information systems due to unreliable or uncertain data [25,26,27]. 

Sound and reliable data quality assessment is thus vital to obtain the high data quality which enhances users’ confidence in public health authorities and their performance [19,24]. As countries monitor and evaluate the performance and progress of established public health indicators, the need for data quality assessment in PHIS that store the performance-and-progress-related data has never been greater [24,28,29]. Nowadays, data quality assessment that has been recommended for ensuring the quality of data in PHIS becomes widespread acceptance in routine public health practice [19,24]. 

Data quality in public health has different definitions from different perspectives. These include: “fit for use in the context of data users” [30], (p. 2); “timely and reliable data essential for public health core functions at all levels of government” [31], (p. 114) and “accurate, reliable, valid, and trusted data in integrated public health informatics networks” [32]. Whether the specific data quality requirements are met is usually measured along a certain number of data quality dimensions. A dimension of data quality represents or reflects an aspect or construct of data quality [33].

Data quality is recognized as a multi-dimensional concept across public health and other sectors [30,33,34,35]. Following the “information chain” perspective, Karr *et al.* used “three hyper-dimensions” (*i.e.*, process, data and user) to group a set of conceptual dimensions of data quality [35]. Accordingly, the methods for assessment of data quality must be useful to assess these three dimensions [35]. We adopted the approach of Karr *et al.* because their typology provided a comprehensive perspective for classifying data quality assessment. However, we replace “process” by “data collection process” and “user” by “data use”. “Process” is a broad term and may be considered as the whole process of data flows, including data and use of data. “User” is a specific term related to data users or consumers and may ignore the use of data. To accurately reflect the data flows in the context of public health, we define the three dimensions of data quality as data, data use and data collection process. The dimension of data focuses on data values or data schemas at record/table level or database level [35]. The dimension of data use, related to use and user, is the degree and manner in which data are used [35]. The dimension of data collection process refers to the generation, assembly, description and maintenance of data [35] before data are stored in PHIS.

**Figure 1:**
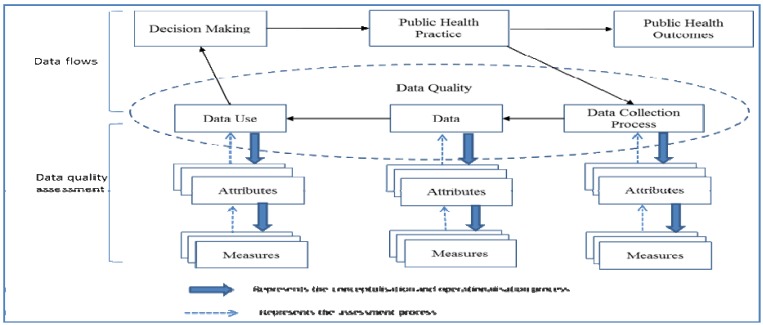
Conceptual framework of data quality assessment in public health practice.

Data quality assessment methods generally base on the measurement theory [35,36,37,38]. Each dimension of data quality consists of a set of attributes. Each attribute characterizes a specific data quality requirement, thereby offering the standard for data quality assessment [35]. Each attribute can be measured by different methods; therefore, there is flexibility in methods used to measure data quality [36,37,38]. As the three dimensions of data quality are embedded in the lifecycle of public health practice, we propose a conceptual framework for data quality assessment in PHIS (Figure 1).

Although data quality has always been an important topic in public health, we have identified a lack of systematic review of data quality assessment methods for PHIS. This is the motivation for this study because knowledge about current developments in methods for data quality assessment is essential for research and practice in public health informatics. This study aims to investigate and compare the methods for data quality assessment of PHIS so as to identify possible patterns and trends emerging over the first decade of the 21st century. We take a qualitative systematic review approach using our proposed conceptual framework.

## 2. Methods

### 2.1. Literature Search

We identified publications by searching several electronic bibliographic databases. These included Scopus, IEEE Xplore, Web of Science, ScienceDirect, PubMed, Cochrane Library and ProQuest. Because many public health institutes also published guidelines, frameworks, or instruments to guide the institutional approach to assess data quality, some well-known institutions’ websites were also reviewed to search for relevant literature. The following words and MeSH headings were used individually or in combination: “data quality”, “information quality”, “public health”, “population health”, “information system *”, “assess *”, “evaluat *”. (“*” was used to find the variations of some word stems.) The articles were confined to those published in English and Chinese language.

The first author performed the literature search between June 2012 and October 2013. The inclusion criteria were peer-refereed empirical studies or institutional reports of data quality assessment in public health or PHIS during the period 2001–2013. The exclusion criteria were narrative reviews, expert opinion, correspondence and commentaries in the topic area. To improve coverage, a manual search of the literature was conducted to identify papers referenced by other publications, papers and well-known authors, and papers from personal databases.

### 2.2. Selection of Publications

**Figure 2:**
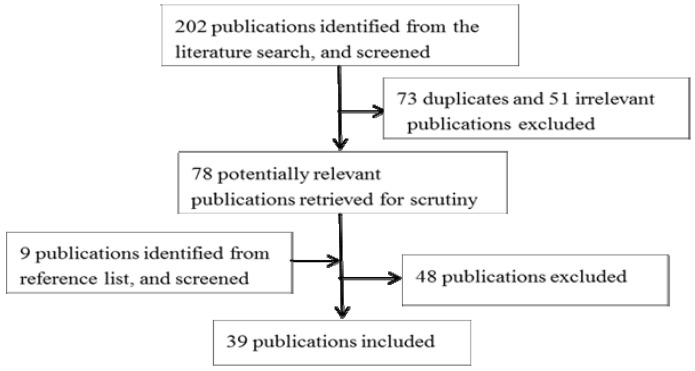
Publication search process.

Citations identified in the literature search were screened by title and abstract for decisions about inclusion or exclusion in this review. If there was uncertainty about the relevance of a citation, the full-text was retrieved and checked. A total of 202 publications were identified and were manually screened. If there was uncertainty about whether to include a publication, its relevance was checked by the fourth author. Finally 39 publications that met the inclusion criteria were selected. The screening process is summarized in Figure 2.

### 2.3. Data Abstraction

The selected publications were stored in an EndNote library. Data extracted from the publications included author, year of publication, aim of data quality assessment, country and context of the study, function and scope of the PHIS, definition of data quality, methods for data quality assessment, study design, data collection methods, data collected, research procedure, methods for data analysis, key findings, conclusions and limitations.

The 39 publications were placed in two groups according to whether they were published by a public health institution at national or international level or by individual researchers. If the article was published by the former, it is referred to as an institutional publication, if by the latter, as a research paper.

## 3. Results

Of the 39 publications reviewed, 32 were peer-refereed research papers and seven were published by public health institutions. The institutional publications are listed in Table 1.

**Table 1:** Institutional data quality assessment publications.

Acronym	Title	Institution
CDC’s Guidelines [15]	Updated Guidelines for Evaluating Public Health Surveillance Systems	United States Centers for Diseases Control and Prevention
CIHI DQF [30]	CIHI Data Quality Framework	Canadian Institute for Health Information
ME DQA [34,39] *	Data Quality Audit Tool	MEASURE Evaluation Project
ME PRISM [40,41]	Performance of Routine Information System Management Version 3.1	MEASURE Evaluation Project
WHO DQA [42,43]	The Immunization Data Quality Audit (DQA)Procedure; Immunization Data Quality Self-assessment (WHO DQS) Tool	Department of Immunization Vaccines and Biologicals, World Health Organization
WHO DQRC [44]	Guide to the Health Facility Data Quality Report Card	World Health Organization
WHO HMN [45]	Assessing the National Health Information System An Assessment Tool Version 4.00	Health Metrics Network, World Health Organization

* ME DQA is adopted by the Global Fund to Fight AIDS, Tuberculosis and Malaria.

27 of the 39 reviewed publications were published between 2008 and 2013. There was a trend of increasing numbers of research papers per year, suggesting an increasing research focus on data quality with the wider adoption of computerised PHIS in recent years. 

The results are organized as follows. First, the aims of the studies are given. This is followed by context and scope identified in Section 3.2. Section 3.3 examines the methods for data quality assessment. A detailed summary of the findings concludes the results in Section 3.4. For each section, a comparison between institutional publications and research papers was conducted, where this was possible and meaningful.

### 3.1. Aims of the Studies

The main aims of the studies are assessing the quality of data (19 publications [30,34,42,44,46,47,48,49,50,51,52,53,54,55,56,57,58,59,60]) and assessing the performance of the PHIS (17 publications [15,22,34,40,42,45,50,58,61,62,63,64,65,66,67,68,69]). Five studies assessed data use and explored the factors influencing data use [26,27,52,70,71]. Four studies investigated the facilitators and barriers for achieving high quality data and systems [22,40,59,65]. Three studies compared or developed methods for the improvement of data quality assessment or data exchange [54,56,72]. Finally two studies assessed data flow [30,70].

The institutions tended to focus on the PHIS system and the data [15,30,34,40,42,44,45]. Data use, comparison of different PHIS, identification of the factors related to poor data quality, and analysis of data flow were also reported in research papers [22,26,27,52,54,56,59,61,65,70,71,72,73].

### 3.2. Context and Scope of the Studies

The contexts of the studies were primarily confined to the public health domain, with other settings addressed occasionally. 

Two types of public health context were covered in the institutional publications. The first included specific disease and health events, such as AIDS, tuberculosis, malaria, and immunization [15,34,42]. The latter was the public health system. This included public health project/program data management and reporting, routine health information systems, and PHIS under a national health institute [34,40,41,44,45]. 

Most research studies were conducted in disease-specific public health contexts. Ten were in the maternal and children’s health setting, e.g., immunization, childbirth, maternal health and hand-foot-mouth disease [47,53,56,57,58,68,69,70,72,73]. Another five were delivered in the context of HIV/AIDS prevention and care [48,49,63,65,67]. Two studies were related to tuberculosis [46,61]. Other contexts included multi-disease surveillance system, primary health care, acute pesticide poisoning, road data or road safety, aboriginal health, monkey pox, and cancer [22,26,51,52,55,59,66,74]. In addition, clinical information management was studied in four research papers [50,54,62,71]. National health management information systems were studied in one publication [27]. 

The public health data from information systems operated by agencies other than public health were also assessed. They include the National Coronial Information System managed by the Victorian Department of Justice in Australia, women veteran mortality information maintained by the U.S. Department of Veterans’ Affairs, and military disability data from U.S. Navy Physical Evaluation Board [47,52,64]. 

The studies were conducted at different levels of the PHIS, including health facilities that deliver the health service and collect data (e.g., clinics, health units, or hospitals), and district, provincial and national levels where PHIS data are aggregated and managed. The institutions took a comprehensive approach targeting all levels of PHIS [15,30,34,40,42,44,45]. Twenty-seven research studies were conducted at a single level [22,26,46,47,48,49,50,51,52,53,54,55,56,57,59,61,62,63,64,66,68,69,70,71,72,73,74]. Of these, 14 were conducted at data collection and entry level. The other 13 studies assessed the PHIS at management level. Only five research papers covered more than one level of the system [27,58,60,65,67], two of which were multi-country studies [58,67]. Lin *et al.* studied the surveillance system at national level, provincial level, and at surveillance sites [65].

### 3.3. Methods for Data Quality Assessment

Analysis of methods for data quality assessment in the reviewed publications is presented in three sections, based on the dimensions of data quality that were covered: data, data use or data collection process. Seven perspectives were reviewed, including quality attributes for each dimension, major measurement indicators for each attribute, study design/method of assessment, data collection methods, data analysis methods, contributions and limitations.

#### 3.3.1. Methods for Assessment of the Dimension of Data

In this section, the concept of data quality is a narrow one, meaning the quality of the dimension of data. All of the institutional publications and 28 research papers, a total of 35 articles, conducted assessment of the quality of data [15,22,30,34,40,42,44,45,46,47,48,49,50,51,52,53,54,55,56,57,58,59,60,61,62,63,64,65,66,67,68,69,72,73,74]. Matheson *et al.* introduced the attributes of data quality but did not give assessment methods [71]. Additional information is provided in Table A1.

##### Quality Attributes of Data and Corresponding Measures

A total of 49 attributes were used in the studies to describe data quality, indicating its multi-dimensional nature. Completeness, accuracy and timeliness were the three attributes measured most often.

Completeness was the most-used attribute of data quality in 24 studies (5 institutional and 19 research publications) [15,22,34,40,42,44,46,48,49,50,51,54,57,61,62,63,64,65,66,68,69,72,73,74]. This was followed by accuracy, in 5 institutional and 16 research publications [15,30,34,40,42,46,48,49,50,51,52,53,56,57,58,63,64,65,69,72,74]. The third most-used attribute, timeliness, was measured in 5 institutional and 4 research publications [22,30,40,42,44,45,64,69,73]. 

The attributes of data quality are grouped into two types: those of good data quality and those of poor data quality (see Table 2).

**Table 2:** Attributes of data quality.

Item	Attribute
High data quality (38)	Completeness, accuracy or positional accuracy, timeliness or up-datedness or currency, validity, periodicity, relevance, reliability, precision, integrity, confidentiality or data security, comparability, consistency or internal consistency or external consistency, concordance, granularity, repeatability, readily useableness or usability or utility, objectivity, ease with understanding, importance, reflecting actual sample, meeting data standards, use of standards, accessibility, transparency, representativeness, disaggregation, data collection method or adjustment methods or data management process or data management
Poor data quality (11)	Missing data, under-reporting, inconsistencies, data errors or calculation errors or errors in report forms or errors resulted from data entry, invalid data, illegible hand writing, non-standardization of vocabulary, and inappropriate fields

Inconsistencies in the definition of attributes were identified. The same attribute was sometimes given different meanings by different researchers. One example of this was “completeness”. Some institutions required conformity to the standard process of data entry, such as filling in data elements in the reporting forms [15,40,41,44]. Completeness was represented as the percentage of blank or unknown data, not zero/missing, or proportion of filling in all data elements in the facility report form [15,40,41,44]. The ME PRISM, instead, defined completeness as the proportion of facilities reporting in an administrative area [40]. The other definition of completeness was the correctness of data collection methods in ME DQA, *i.e.*, “complete list of eligible persons or units and not just a fraction of the list” [34].

Of the 19 research papers including completeness as an attribute, 12 measured the completeness of data elements as “no missing data or blank” [22,46,48,49,50,51,57,63,69,72,73,74]. Dixon *et al.* defined completeness as considering both filling in data elements and data collection methods [54]. Four studies measured completeness of data by the sample size and the percentage of health facilities that completed data reports [61,65,66,68]. The remaining two studies did not give precise definitions [51,64]. 

On the other hand, different attributes could be given the same meaning. For example, the ME DQA defined accuracy as “validity”, which is one of two attributes of data quality in CDC’s Guidelines [15,34]. Makombe *et al.* considered that data were accurate if none of the examined variables in the site report was missing [49]. This is similar to the definition of completeness, as “no missing data” or “no blank of data elements” in the reports by other studies.

##### Study Design

Quantitative methods were used in all studies except that of Lowrance *et al.* who used only qualitative methods [63]. Retrospective, cross-sectional survey was commonly used for quantitative studies. Pereira *et al.* conducted a multi-center randomized trial [72].

Qualitative methods, including review of publications and documentations, interviews with key informants, and field observations, were also used in 8 studies [34,45,50,57,61,65,69,72]. The purpose of the application of qualitative methods was primarily to provide the context of the findings from the quantitative data. For example, Hahn *et al.* conducted a multiple-case study in Kenya to describe clinical information systems and assess the quality of data. They audited a set of selected data tracer items, such as blood group and weight, to assess data completeness and accuracy. Meanwhile, they obtained end-users’ views of data quality from structured interviews with 44 staff members and qualitative in-depth interviews with 15 key informants [50].

The study subjects varied. In 22 publications, the study subjects were entirely data [15,42,44,46,47,48,49,51,52,53,54,55,56,58,59,60,64,66,67,68,73,74]; in four of these publications, they were entirely users or stakeholders of the PHIS [30,45,62,63]. Three publications studied both the data and the users [22,50,72]. Study subjects in research included data and documentations by Dai *et al.* [69]; data, documentation of instructions, and key informants in four studies [34,40,57,61]; and data, user, documentations of guidelines and protocols, and the data collection process by Lin *et al.* [65]. Both data and users as study subjects were reported in eight publications [22,34,40,50,57,61,65,72]. 

The sampling methods also varied. Only the study by Clayton *et al.* calculated sample size and statistical power [56]. Freestone *et al.* determined the sample size without explanation [52]. One study used two-stage sampling [56]. Ten studies used multi-stage sampling methods [22,34,42,48,52,55,56,58,68,72]. The rest used convenience or purposive sampling. The response rates were reported in two studies [62,72].

The data collection period ranged from one month to 16 years [67,74]. The study with the shortest time frame of one month had the maximum number of data records, 7.5 million [67], whereas the longest study, from 1970 to 1986, collected only 404 cases of disease [74]. The sample size of users ranged from 10 to 100 [45,61].

##### Data Collection Methods

Four methods were used individually or in combination in data collection. These were: field observation, interview, structured and semi-structured questionnaire survey, and auditing the existing data. Field observation was conducted using checklist and rating scales, or informal observations on workplace walkthroughs [34,40,50,65]. Open, semi-structured or structured interviews were used when the study subjects were users or stakeholders of the PHIS [30,40,45,50,57,61,62,63,65]. Auditing was used in directly examining existing datasets in PHIS, looking for certain data elements or variables. The benchmarks used for auditing included: in-house-defined data standards, international or national gold standards, and authoritative datasets [15,40,42,44,46,48,49,51,52,53,54,55,56,58,59,64,66,67,68,72,73,74]. The effect of auditing was enhanced by field observations to verify the accuracy of data sets [34,40,42,50,58,65]. 

##### Data Analysis Methods

Data analysis methods were determined by the purpose of the study and the types of data collected.

For the quantitative data, descriptive statistics were often used. For example, continuous data were usually analyzed by the value of percentage, particularly for the data about completeness and accuracy, to ascertain whether they reached the quality standards. This method was most often used in 24 papers [22,34,40,42,44,46,47,48,49,50,52,53,54,55,56,57,58,59,64,65,66,68,72,73]. Plot chart, bubble scatter chart, and confidence intervals were also used in two studies [52,68]. Other common statistical techniques included: correlation relationship, the Chi-square test, and the Mann–Whitney test [56,58,68]. The geographic information system technique was reported in 3 studies [51,52,74]. Seven studies reported the use of questionnaires or checklists with a Likert scale or a yes/no tick, as well as simple, summative and group scoring methods [30,34,40,45,58,61,62]. 

In the publications with data as the study subject, a certain number of data variables were selected, but the reason(s) for the section was (were) not always given. They included elements of demographics such as age, gender, and birth date, and specific information such as laboratory testing results, and disease code. The minimum and maximum number of data variables was 1 and 30, respectively [58,59]. 

The qualitative data were transcribed first before semantic analysis by theme grouping methods [63].

#### 3.3.2. Methods for Assessment of the Dimension of Data Use

Ten studies, including one institutional publication and nine research papers, are reviewed in this section [26,27,40,45,50,52,61,62,70,71]. Five studies were concerned with the assessment of data use and the factors influencing data use [26,27,52,70,71]. The other five included assessment of data use, but this was not always highlighted [40,45,50,61,62]. Details are given in Table A2.

##### Quality Attributes of Data Use and Corresponding Measures

A total of 11 attributes were used to define the concept of data use. These were: trend in use, use of data or use of information, system use or usefulness of the system, intention to use, user satisfaction, information dissemination or dissemination of data, extent of data source recognition and use or specific uses of data, and existence and contents of formal information strategies and routines. 

The measures fall into three categories: data use for the purpose of action, planning and research; strategies and mechanisms of data use; and awareness of data sources and data use. 

The first category of measures was mentioned in eight studies [26,40,45,50,52,61,70,71]. For example, actioned requests from researchers, the number of summaries/reports produced, and the percentage of report use [40,52,71]. Freestone *et al.* calculated actioned requests from researchers who do not have access to the PHIS [52]. The measurement indicators in ME PRISM were report production and display of information. They were assessed by whether and how many reports containing data from the PHIS were compiled, issued, fed back and displayed for a set time frame [40]. Saeed *et al.* assessed the use of data by predefined criteria, including the availability of comprehensive information, whether data were used for planning and action at each level, and whether feedback was given to the lower organizational level of the public health system [61]. 

The second category of measures was assessed in five studies [26,27,45,61,70]. The criteria of the measurement included the availability of a feedback mechanism, policy and advocacy, the existence and the focus of formal information strategies, and routines of data use [26,45,70]. 

The third category measured users’ awareness of data use which was reported in two studies [26,62]. Petter and Fruhling applied the DeLone and McLean information systems success model [62]. They used the framework to evaluate system use, intention to use, and user satisfaction in 15 questions by considering the context of the PHIS, which was an emergency response medical information system. Wilkinson and McCarthy recommended examining whether the studied information systems were recognized by the users in order to assess the extent of data source recognition among respondents [26].

##### Study Design

Three studies only used quantitative methods [40,52,62] and three studies only used qualitative methods [27,50,70]. The remaining four studies combined qualitative and quantitative methods [26,45,61,71]. Interviews, questionnaire surveys, reviews of documentation and abstracts of relevant data were used in the studies. 

##### Data Collection Methods

The sources of information for the study subjects included users and stakeholders, existing documents, and data from the PHIS. Study subjects were all users in six studies [26,27,45,50,62,70], and all data in the study by Freestone *et al.* [52]. Both user and documentation were study subjects in two studies [40,61], and together with data in another study [71]. Convenience or purposive sampling was generally used. 

Among nine studies whose study subjects were users, structured and semi-structured questionnaire surveys, group discussions, and in-depth interviews were used to collect data. Use of self-assessment, face-to-face communication, telephone, internet telephony, online, email, facsimile and mail were reported in the studies. For example, Wilkinson and McCarthy used a standardized semi-structured questionnaire for telephone interviews with key informants [26]. Petter and Fruhling used an online survey as well as facsimile and mail to the PHIS users [62]. Qazi and Al administered in-depth, face-to-face and semi-structured interviews with an interview guide [27]. Saeed *et al.* predefined each criterion for data use and measured it by a 3-point Likert scale. They assessed each criterion through interviewing key informants and consulting stakeholders*.* Desk review of important documents, such as national strategic plans, guidelines, manuals, annual reports and databases was also reported in their study [61]. 

Four studies assessing data use by data and documentation either queried information directly from the data in the studied PHIS, if applicable, or collected evidence from related documents such as reports, summaries, and guidelines [40,52,61,71]. The data to be collected included actioned requests, the number of data linked to action, and the number of data used for planning. Time for data collection varied without explanation, such as 12 months in ME PRISM or six years by Freestone *et al.* [40,52].

##### Data Analysis Methods

The data collected from qualitative studies were usually processed manually, organized thematically or chronologically. They were either analyzed by classification of answers, grouping by facility or respondent’s role, or categorization of verbatim notes into themes. 

Various strategies were applied for quantitative data. For example, Wilkinson and McCarthy counted the same or similar responses to indicate frequency of beliefs/examples across participants [26]. Data in their study were analyzed individually, by role and aggregated level. Some correlational analyses, such as Pearson’s r for parametric data and Spearman’s Rho for non-parametric data, were conducted to identify possible relationships between data use, perceptions of data, and organizational factors. Petter and Fruhling conducted hypothesis analysis in structured questionnaire with a 7-point Likert scale for all quantitative questions [62]. Due to the small sample size of 64 usable responses, they used summative scales for each of the constructs. All of the items used for a specific construct were averaged to obtain a single value for this construct. Then, using this average score, each hypothesis was tested using simple regression.

#### 3.3.3. Methods for Assessment of the Dimension of Data Collection Process

Although the aim of assessing data flow or the process of data collection was only stated in two studies, another 14 articles were found that implicitly assessed data collection process [22,30,34,40,42,45,50,52,55,58,59,60,65,67,69,70]. These articles were identified through a detailed content analysis. For example, data collection process assessment activities were sometimes initiated by identification of the causes of poor data quality [52,55,59]. Or data collection process was considered as a component of the evaluation of the effectiveness of the system [22,34,42,45,58,60,65,69]. Three studies led by two institutions, CIHI and MEASURE Evaluation Project, assessed data collection process while conducting assessment of the quality of the data, [30,40,50]. Details are given in Table A3.

##### Quality Attributes of Data Collection Process and Corresponding Measures

A total of 23 attributes of data collection process were identified. These were: quality index or quality scores or functional areas, root causes for poor data quality, metadata or metadata documentation or data management or case detection, data flow or information flow chart or data transmission, data collection or routine data collection or data recording or data collection and recording processes or data collection procedures, data quality management or data quality control, statistical analysis or data compilation or data dissemination, feedback, and training.

Only four studies explicitly defined the attributes of the dimension of data collection process, two of them from institutions [40,45,52,70]. Data collection was the most-used attribute in six publications [34,40,52,65,67,69,70]. The next most-assessed attribute is data management processes or data control reported in four publications [34,45,67,69]. 

Data collection process was sometimes considered a composite concept in six studies, four of them proposed by institutions [30,34,42,45,58,60]. For example, the quality index/score was composed of five attributes: recording practices, storing/reporting practices, monitoring and evaluation, denominators, and system design (the receipt, processing, storage and tabulation of the reported data) [42,58,60]. Metadata documentation or metadata dictionary cover dataset description, methodology, and data collection, capture, processing, compilation, documentation, storage, analysis and dissemination [30,45]. The ME DQA assessed five functional areas, including structures, functions and capabilities, indicator definitions and reporting guidelines, data collection and reporting forms and tools, data management processes, and links with the national reporting system [34].

##### Study Design

Seven studies only used qualitative methods [50,52,55,59,65,69,70], five only conducted quantitative research [22,30,40,58,67], and four used both approaches [34,42,45,60]. Questionnaire surveys were reported in 10 papers [22,30,34,40,42,45,58,60,67,70]. Interviews were conducted in 3 studies [34,50,70]. Focus group approaches, including consultation, group discussion, or meeting with staff or stakeholders, were reported in four studies [45,52,59,65]. Review of documentation was conducted in five papers [34,40,52,55,69], and field observation was used in five studies [34,40,50,52,65].

##### Data Collection and Analysis Methods

The study subjects included managers or users of the PHIS, the documentation of instructions and guidelines of data management for the PHIS, and some procedures of data collection process. The study subjects were entirely users in eight studies [22,30,40,45,58,59,67,70]. Corriols *et al.* and Dai *et al.* only studied documentation such as evaluation reports on the PHIS including deficiency in the information flow chart and non-reporting by physicians [55,69]. Data collection process was studied in six publications [34,45,50,52,60,65]. Of these, four studies combined data collection procedures with users and documentation [34,42,52,65], while Hahn *et al.* only observed data collection procedures and Ronveaux *et al.* surveyed users and observed data collection procedures for a hypothetical population [50,60].

The data collection methods included field observation, questionnaire surveys, consensus development, and desk review of documentation. Field observations were conducted either in line with a checklist or in an informal way [34,40,50,52,60,65]. Lin *et al.* made field observations of the laboratory staff dealing with specimens and testing at the early stage of the data collection process [65]. Freestone *et al.* observed data coders’ activities during the process of data geocoding and entry [52]. Hahn *et al.* followed the work-through in study sites [50]. WHO DQA conducted field observations on sites of data collection, processing and entry [42], while Ronveaux *et al.* observed workers at the health-unit level who completed some data collection activities for 20 hypothetical children [60]. ME DQA made follow-up on-site assessment of off-site desk-reviewed documentation at each level of the PHIS [34]. 

Questionnaire surveys included semi-structured and structured ones [22,30,34,40,42,45,58,60,67,70]. The questionnaire data were collected by face-to-face interviews, except one online questionnaire survey study by Forster *et al.* [67]. Five studies used a multi-stage sampling method [22,34,42,58,60]. The rest surveyed convenience samples or samples chosen according to a particular guideline, which was sometimes not described [30,34,40]. 

Consensus development was mainly used in group discussion and meetings, guided by either structured questionnaires or data quality issues [45,59]. Ancker *et al.* held a series of weekly team meetings over about four months with key informants involved in data collection [59]. They explored the root causes of poor data quality in line with the issues identified from assessment results. WHO HMN organized group discussions with approximately 100 major stakeholders [45]. Five measures related to data collection process were contained in a 197-item questionnaire. The consensus to each measure was reached through self-assessment, individual or group scoring to yield a percentage rating [45]. 

Desk review of documentation was reported in six studies [34,52,55,65,69,70]. The documentation included guidelines, protocols, official evaluation reports and those provided by data management units. The procedures for appraisal and adoption of relevant information were not introduced in the studies. 

Data analysis methods for quantitative studies were mainly descriptive statistics. Most papers did not present the methods for analysis of the qualitative data. Information retrieved from the qualitative study was usually triangulated with findings from quantitative data.

### 3.4. Summary of the Findings

Four major themes of the results have emerged after our detailed analysis, which are summarized in this section. 

The first theme is there are differences between the seven institutional and the 32 individual research publications in their approach to data quality assessment, in terms of aims, context and scope. First, the effectiveness of the PHIS was more of an institutional rather than a researcher’s interest. It was covered in all of the institutional publications but only in one-third of the research papers. Second, the disease-specific public health contexts covered by United Nations’ MDGs, maternal health, children’s health, and HIV/AIDS, were the area most often studied by researchers. Whereas the institutions also paid attention to the routine PHIS. Third, the institutions tended to evaluate all levels of data management whereas most research studies were focused on a single level of analysis, either record collection or management.

The second theme is coverage of the three dimensions of data quality was not equal. The dimension of data was most frequently assessed (reported in 35 articles). Data use was explicitly assessed in five studies and data collection process in one. Implicit assessment of data use and data collection process was found in another five and 15 papers, respectively. The rationale for initiating these implicit assessments was usually to identify factors arising from either data use or data collection process while assessing the quality of data. Within studies that considered more than one dimension of data quality, 15 assessed both data and data collection process, seven assessed data and data use and one, both data use and data collection process. Only four studies assessed all three dimensions of data quality. 

The third emerging theme is a lack of clear definition of the attributes and measurement indicators of each dimension of data quality. First, a wide variation of the definition of the key terms was identified, including the different terms for the same attribute, and the same term to refer to distinct attributes. The definition of attributes and their associated measures was sometimes given based on intuition, prior experience, or the underlying objectives unique to the PHIS in a specific context. 

Second, the attributes of the quality of data were relatively developed than those for the dimensions of data use and data collection process. Most definitions of data quality attributes and measures are referred to the dimension of data as opposed to the other two dimensions, the attributes of which were primarily vague or obscure. One clear gap is the absence of the attributes of the dimension of data collection process.

Third, a consensus has not been reached as to what attributes should be measured. For example, a large variety existed in the number of attributes measured in the studies varied between 1 and 8, in a total of 49 attributes. The attribute of data quality in public health is often measured positively in terms of what it is. The three most-used attributes of good data quality were completeness, accuracy, and timeliness. The institutions tended to assess more attributes of data quality than individual researchers. The number of attributes reported in research papers was no more than four, while the institutions assessed at least four attributes. 

The last emerging theme of the results is methods of assessment lack systematic procedures. Quantitative data quality assessment primarily used descriptive surveys and data audits, while qualitative data quality assessment methods include primarily interview, documentation review and field observation. Both objective and subjective strategies were identified among the methods for assessing data quality. The objective approach applies quantifiable measurements to directly examine the data according to a set of data items/variables/elements/tracer items. The subjective approach measures the perceptions of the users and stakeholders of the PHIS. However, only a small minority of the reviewed studies used both types of assessment. Meanwhile, field verification of the quality of data is not yet a routine practice in data quality assessment. Only five studies conducted field observations for data or for data collection process and they were usually informal. The reliability and validity of the study was rarely reported. 

## 4. Discussion

Data are essential to public health. They represent and reflect public health practice. The broad application of data in PHIS for the evaluation of public health accountability and performance has raised the awareness of public health agencies of data quality, and of methods and approaches for its assessment. We systematically reviewed the current status of quality assessment for each of the three dimensions of data quality: data, data collection process and data use. The results suggest that the theory of measurement has been applied either explicitly or implicitly in the development of data quality assessment methods for PHIS. The majority of previous studies assessed data quality by a set of attributes using certain measures. Our findings, based on the proposed conceptual framework of data quality assessment for public health, also identified the gaps existed in the methods included in this review. 

The importance of systematic, scientific data quality assessment needs to be highlighted. All three dimensions of data quality, data, data use and data collection process, need to be systematically evaluated. To date, the three dimensions of data quality were not given the same weight across the reviewed studies. The quality of data use and data collection process has not received adequate attention. This lack of recognition of data use and data collection process might reflect a lack of consensus on the dimensions of data quality. Because of the equal contributions of these three dimensions to data quality, they should be given equal weight in data quality assessment. Further development in methods to assess data collection process and data use is required. 

Effort should also be directed towards clear conceptualisation of the definitions of the relevant terms that are commonly used to describe and measure data quality, such as the dimensions and attributes of data quality. The lack of clear definition of the key terms creates confusions and uncertainties and undermines the validity and reliability of data quality assessment methods. An ontology-based exploration and evaluation from the perspective of data users will be useful for future development in this field [33,75]. Two steps that involve conceptualization of data quality attributes and operationalization of corresponding measures need to be taken seriously into consideration and rationally followed as shown in our proposed conceptual framework. 

Data quality assessment should use mixed methods (e.g., qualitative and quantitative assessment methods) to assess data from multiple sources (e.g., records, organisational documentation, data collection process and data users) and used at different levels of the organisation [33,35,36,38,75,76]. More precisely, we strongly suggest that subjective assessments of end-users’ or customers’ perspectives be an indispensible component in data quality assessment for PHIS. The importance of this strategy has long been articulated by the researchers [33,75,76]. Objective assessment methods assess the data that were already collected and stored in the PHIS. Many methods have been developed, widely accepted and used in practice [38,76]. On the other hand, subjective assessments provide a supplement to objective data quality assessment. For example, interview is useful for the identification of the root causes of poor data quality and for the design of effective strategies to improve data quality. Meanwhile, field observation and validation is necessary wherever it is possible because reference of data to the real world will give data users confidence in the data quality and in application of data to public health decision-making, action, and outcomes [52]. The validity of a study would be doubtful if the quality of data could not be verified in the field [36], especially when the data are come from a PHIS consisting of secondary data. 

To increase the rigor of data quality assessment, the relevant statistical principles for sample size calculation, research design, measurement and analysis need to be adhered to. Use of convenience or specifically chosen sampling methods in 24 studies included in this review reduced the representativeness and generalizability of the findings of these studies. At the same time, reporting of data quality assessment needs to present the detailed procedures and methods used for the study, the findings and limitations. The relatively simple data analysis methods using only descriptive statistics could lead to loss of useful supportive information. 

Finally, to address the gaps identified in this review, we suggest re-prioritizing the orientation of data quality assessment in future studies. Data quality is influenced by technical, organizational, behavioural and environmental factors [35,41]. It covers large information systems contexts, specific knowledge and multi-disciplinary techniques [33,35,75]. Data quality in the reviewed studies is frequently assessed as a component of the quality or effectiveness or performance of the PHIS. This may reflect that the major concern of public health is in managerial efficiency, especially of the PHIS institutions. Also, this may reflect differences in the resources available to, and the responsibilities of institutions and individual researchers. However, data quality assessment hidden within other scopes may lead to ignorance of data management and thereby the unawareness of data quality problems enduring in public health practice. Data quality needs to be positioned at the forefront of public health as a distinct area that deserves specific scientific research and management investment. 

While this review provides a detailed overview of data quality assessment issues, there are some limitations in its coverage, constrained by the access to the databases and the breadth of public health information systems making it challenge to conduct systematic comparison among studies. The search was limited by a lack of subject headings for data quality of PHIS in MeSH terms. This could cause our search to miss some relevant publications. To compensate for this limitation, we used the strategy of searching well-known institutional publications and manually searching the references of each article retrieved. 

Our classification process was primarily subjective. It is possible that some original researchers disagree with our interpretations. Each assessment method has contributions and limitations which make the choices difficult. We provided some examples of approaches to these issues.

In addition, our evaluation is limited by an incomplete presentation of details in some of the papers that we reviewed. A comprehensive data quality assessment method includes a set of guidelines and techniques that defines a rational process to assess data quality [37]. The detailed procedure of data analysis, data quality requirements analysis, and identification of critical attributes is rarely given in the reviewed papers. A lack of adequate detail in the original studies could have affected the validity of some of our conclusions. 

## 5. Conclusions

Public health is a data-intensive field which needs high-quality data to support public health assessment, decision-making and to assure the health of communities. Data quality assessment is important for public health. In this review of the literature we have examined the data quality assessment methods based on our proposed conceptual framework. This framework incorporates the three dimensions of data quality in the assessment methods for overall data quality: data, data use and data collection process. We found that the dimension of the data themselves was most frequently assessed in previous studies. Most methods for data quality assessment evaluated a set of attributes using relevant measures. Completeness, accuracy, and timeliness were the three most-assessed attributes. Quantitative data quality assessment primarily used descriptive surveys and data audits, while qualitative data quality assessment methods include primarily interview, documentation review and field observation. 

We found that data-use and data-process have not been given adequate attention, although they were equally important factors which determine the quality of data. Other limitations of the previous studies were inconsistency in the definition of the attributes of data quality, failure to address data users’ concerns and a lack of triangulation of mixed methods for data quality assessment. The reliability and validity of the data quality assessment were rarely reported. These gaps suggest that in the future, data quality assessment for public health needs to consider equally the three dimensions of data quality, data, data use and data process. More work is needed to develop clear and consistent definitions of data quality and systematic methods and approaches for data quality assessment. 

The results of this review highlight the need for the development of data quality assessment methods. As suggested by our proposed conceptual framework, future data quality assessment needs to equally pay attention to the three dimensions of data quality. Measuring the perceptions of end users or consumers towards data quality will enrich our understanding of data quality issues. Clear conceptualization, scientific and systematic operationalization of assessment will ensure the reliability and validity of the measurement of data quality. New theories on data quality assessment for PHIS may also be developed. 

## Appendix

**Table A1:** Characteristics of methods for assessment of the data dimension reported in the 36 publications included in the review.

Authors Year	Attributes Major measures	Study design	Data collection methods	Data analysis methods	Contribution	Limitations
Ancker *et al.* 2011 [59]	Percentage of missing data, inconsistencies and potential errors of different variables; number of duplicate records, number of non-standardization of vocabulary, number of inappropriate fields	Quantitative audit of data attributes of dataset.	Selected one data set and used tools to query 30 variables, manually assessed data formats	Rates, percentage or counts	Identified data quality issues and their root causes.	Need a specific data query tool
Bosch-Capblanch * et al.* 2009 [58]	Accuracy Proportions in the relevant data set, such as the recounted number of indicator’s data by the reported number at the next tier in the reporting system. A ratio less than 100% indicates “over-reporting”; a ratio over 100% suggests “under-reporting”	Quantitative audit of data accuracy by external auditors applying WHO DQA in 41 countries	A multistage weighted representative random sampling procedure, field visits verifying the reported data. Compared data collected from fields with the reports at the next tier	Percentage, median, inter-quartile range, 95% confidence intervals, ratio (verification factor quotient) adjusted and extrapolated	Systematic methodology to describe data quality and identify basic recording and reporting practices as key factors and good practices	Limited attributes, lack of verification of source of actual data and excluded non-eligible districts
CDC 2001 [15]	Completeness, accuracy Percentage of blank or unknown responses, ratio of recorded data values over true values	Quantitative audit of dataset, a review of sampled data, a special record linkage, or a patient interview	Calculating the percentage of blank or unknown responses to items on recording forms, reviewing sampled data, conducting record linkage, or a patient interview	Descriptive statistics: percentage	Provides generic guidelines	Lack of detail on procedures, needs adjustment
Chiba *et al.* 2012 [57]	Completeness: percentage of complete data. Accuracy: 1-percentage of the complete data which were illegible, wrongly coded, inappropriate and unrecognized. Relevance: comparing the data categories with those in upper level report to evaluate whether the data collected satisfied management information needs	Quantitative verification of data accuracy and completeness, and qualitative verification of data relevance in a retrospective comparative case study	Purposive sampling, clinical visits, re-entered and audited 30 data categories of one year data to evaluate accuracy and completeness; qualitatively examined data categories and instructions to assess the relevance, completeness and accuracy of the data, semi-structured interviews to capture factors that influence data quality	Descriptive statistics for accuracy and completeness of the data. Qualitative data were thematically grouped and analyzed by data categories, instructions, and key informants’ views	Quantitative and qualitative verification of data quality; comparison of two hospitals increased generalizability of the findings	Consistency and timeliness were not assessed. Data from the system were not able to be validated
CIHI 2009 [30]	Accuracy: coverage, capture and collection, unit non-response, item (partial) non-response, measurement error, edit and imputation, processing and estimation. Timeliness: data currency at the time of release, documentation currency. Comparability: data dictionary standards, standardization, linkage, equivalency, historical comparability. Usability: accessibility, documentation, interpretability. Relevance: adaptability, value.	Quantitative method, user survey-questionnaire	Questionnaire by asking users, three ratings of each construct, including met, not met, unknown or not applicable (or minimal or none, moderate, significant or unknown) All levels of the system were taken into account in the assessment	Descriptive statistics for ratings by each criterion, the overall assessment for a criterion based on the worst assessment of the applicable levels	Data quality assessed from user’s perspective provides comprehensive characteristics and criteria of each dimension of data quality. 5 dimensions, 19 characteristics and 61criteria	Undefined procedures of survey including sample size. Being an internal assessment, rating scores were used for internal purposes
Clayton *et al.* 2013 [56]	Accuracy Sensitivity, specificity, positive predictive value (PPV), negative predictive value (NPV)	Quantitative method to audit dataset by power calculation of 840 medical records	Two stage sampling of study sites, abstracting records and auditing 25 data variables to assess accuracy of the data reported on three data sources	Descriptive statistics were calculated for each data sources; summary measure of kappa values sing the paired sample Wilcoxon signed rank test	Accessing and linking three data sources—maternal medical charts, birth certificates and hospital discharge data whose access is limited and using the medical chart as the gold standard	Limited generalizability of the findings; low sample size and limited representativeness
Corriols *et al.* 2008 [55]	Under-reporting Calculating the difference between registered cases and surveyed cases	Quantitative method to administer a cross-sectional survey in the country	4 stage consistent random sampling method across the country. Face-to-face interview questionnaire survey.	Descriptive statistics for estimation of national underreporting by using survey results	Good representativeness of the study population	Lack of case diagnosis information and the quality of the source of the data
Dai *et al.* 2011 [69]	Under-reporting, errors on report forms, errors resulted from data entry; completeness of information, accuracy, timeliness	Qualitative and quantitative methods by reviewing publications on the system and data from the system	Reviewing publications on the system and data from the system	Descriptive statistics for quantitative data and thematically grouping for qualitative data	Evaluated all existing sub-systems included in the system	Undefined procedures of review, lack of verification of source data
Authors Year	Attributes Major measures	Study design	Data collection methods	Data analysis methods	Contribution	Limitations
Dixon *et al.* 2011 [54]	Completeness The proportion of diagnosed cases and the proportion of fields in a case report	Quantitative method by auditing dataset	Creating a minimum data set of 18 key data elements, using structured query language (SQL) statements to calculate the percent completeness of each field of a total of 7.5 million laboratory reports	Descriptive statistics to calculate the difference between the completeness scores across samples	Development of a method for evaluating the completeness of laboratory data	Need a specific data query tool and only assessed completeness
Edmond *et al.* 2011 [68]	Completeness, illegible hand writing, calculation errors The proportion of the consultation rates for two items, the proportion of illegible hand writing and required clarification, and the proportion of calculation errors on the submitted record forms	Quantitative method: audit the submitted record forms in the dataset	3303 cards from randomly selected five weeks from each year between 2003 and 2009	Descriptive statistics for the percentage of each data quality attribute	Random selection of dataset	Only calculated completeness, without field verification of accuracy of data
Ford *et al.* 2007 [53]	Accuracy Sensitivity, specificity and positive predictive values	Quantitative method to use record linkage to audit dataset, comparing the system with a gold standard (a statewide audit dataset)	Calculated data quality indicators for 18 data variables, compared with a statewide audit (gold standard), including 2432 babies admitted to NICUs, 1994–1996	Descriptive statistics with exact binomial confidence intervals for data quality attributes, comparing two datasets by using the chi-square test	The findings are consistent with other validation studies that compare routinely collected population health data with medical records	Lack of verification of variations between two datasets, inadequate representativeness
Forster *et al.* 2008 [67]	Missing data The percentage of the missing data	Quantitative method to audit dataset	Assessed data quality of a set of six key variables. A global missing data index was computed determining the median of the percentages missing data. Sites were ranked according to this index	Confidence interval (CI), Conbach’s, multivariate logic models, Spearman rank correlation coefficient	Directly examined associations between site characteristics and data quality	Convenience sample and uncertain generalizability
Freestone *et al.* 2012 [52]	Accuracy, consistency, granularity	Quantitative method to audit dataset from three components: source documents, data extraction/transposition, and data cleaning	Systematic sampling 200 cases, each geocoded and comparatively assessed of data quality with and without the influence of geocoding, by pre-selected criteria	Data quality measured by category: perfect, near perfect, poor. Paired t-test for 200 samples and chi-square test for year	Quantify data quality attributes with different factors	No reference type and no field verification (for historic data)
Frizzelle *et al.* 2009 [51]	Accuracy, completeness, currency Assessed by positional errors, generalizations incompatible with highly accurate geospatial locations, updated with the change	Quantitative method to use geographic information systems (GIS) by developing a custom road dataset for analyzing data quality of four datasets	Developed a custom road dataset, and compared with four readily available public and commercial road datasets; developed three analytical measures to assess the comparative data quality	Percentage, concordance coefficients and Pearson correlation coefficients	Exemplary to assessing the feasibility of readily available commercial or public road datasets and outlines the steps of developing a custom dataset	No field verification for historic data
Hahn *et al.* 2013 [50]	Completeness, accuracy The percentage of correctly or completely transmitted items from the original data source to secondary data sources	A multiple case study by quantitative and qualitative approaches in 3 antenatal care clinics of two private and one public Kenyan hospital	Quantitative method: selected 11 data tracer items followed retrospectively and audited compared to independently created gold standard. Qualitative methods: structured interviews and qualitative in-depth interviews to assess the subjective dimensions of data quality. Five-point scales were used for each statement. Purposeful sampling of 44 staff for survey and 15 staff for key informants interviews	Quantitative data: manual review, descriptive statistics, Kruskal-Wallis test, Mann-Whitney U test for continuous measures. Qualitative data: processed manually and classified and grouped by facility and staff class	Combining different methods and viewing the information systems from different viewpoints, covering the quality of PHIS and drawing suggestions for improvement of data quality from qualitative results, likely to produce robust results in other settings	
Harper *et al.* 2011 [66]	Completeness: the proportion of filled fields on the reports. Validity: the proportion of the number of the written indicators against the assigned standard; the proportion of entered incorrect numbers; the proportion of illegible entries; the proportion of entries out of chronological order	Quantitative method to audit an electronic database that was manually extracted entries of a reference syndrome from anonymized dataset from the E-Book health registry entries	Using a random systematic sample of 10% of the extracted entries (*i.e.*, beginning with a randomly chosen starting point and then performing interval sampling to check 10% of records), with an acceptable error rate of <5%	Descriptive statistics on attributes. To avoid bias, age and sex proportions were extracted from available records, the proportions compared to National Census data.	Examine data quality using a reference syndrome, thus making it possible to provide informed recommendations. Descriptive data analysis provides grounded and useful information for decision makers	No evaluation of data collection methods
Hills *et al.* 2012 [73]	Timeliness: the number of days between Service Date and Entry Date of submission of data to the system (three categories: ≤7 days, =8–30 days, and ≥31 days). Completeness: the complete recording of data elements by calculating the proportion of complete fields over total number of fields	Quantitative method to audit data set	Use a de-identified 757,476 demographic records and 2,634,101 vaccination records from the system	Descriptive statistics on attributes	Large dataset provides a statistically significant association	Not able to examine two highly relevant components of data quality: vaccination record coverage completeness and accuracy
Lash *et al.* 2012 [74]	Completeness: the number of locations matching to latitude and longitude coordinates. Positional accuracy: spatial resolution of the dataset. Concordance: the number of localities falling within the boundary. Repeatability: the georeferencing methodology	Georeferencing historic datasets, quantitative method research historic data with 404 recorded MPX cases in seven countries during 1970–1986 from 231 unique localities	Develop ecological niche models and maps of potential MPX distributions based on each of the three occurrence data sets with different georeferencing efforts	Descriptive statistics on attributes and comparison of georeferencing match rates	Document the difficulties and limitations in the available methods for georeferencing with historic disease data in foreign locations with poor geographic reference information.	Not able to examine the accuracy of data source
Lin *et al.* 2012 [65]	Completeness: sufficient sample size. Accuracy: data missing or discrepancies between questionnaires and database	Quantitative and qualitative methods, auditing data set by cross-checking 5% questionnaires against the electronic database during the field visits	Review guidelines and protocols using a detailed checklist; purposive sampling; direct observations of data collection; cross-checking compared database with the questionnaires	Descriptive statistics for attributes of data quality	Mixed-methods to assess data quality	Unable to generalize the findings to the whole system
Litow and Krahl 2007 [64]	Accuracy, use of standards, completeness, timeliness, and accessibility	Quantitative method based on a framework developed for assessment of PHIS	Exported and queried one year data by 12 data items	Descriptive statistics for data quality attributes	Research on Navy population for public health applicability of the system and identified factors influencing data quality	Needs a framework which was undefined in the research
Lowrance *et al.* 2007 [63]	Completeness, updated-ness, accuracy	Qualitative method by following CDC’s Guidelines with qualitative methods	Standardized interviews with 18 key informants during 12 site visits, and meetings with stakeholders from government, non-governmental and faith-based organizations.	Thematically grouping interview responses	Data quality qualitatively assessed by key informants and stakeholders	Lack of quantifiable information
Authors Year	Attributes Major measures	Study design	Data collection methods	Data analysis methods	Contribution	Limitations
Makombe *et al.* 2008 [49]	Completeness: filled fields; accuracy: no missing examined variables or a difference less than 5% compared to the supervision report	Quantitative methods to audit the quality of site reports as of the date of field supervisory visits	6 case registration fields and 2 outcome data were examined	Descriptive statistics on attributes of data quality from site reported were compared to those of supervision reports (“gold standard”)	Set up thresholds of accuracy, examine association between facility characteristics and data quality	Only assessed aggregated facility-level rather individual patient data
Mate *et al.* 2009 [48]	Completeness: no missing data in a period of time; accuracy: the value in the database was within 10% of the gold standard value or percentage deviation from expected for each data element when compared to the gold standard data set	Quantitative methods to assess attributes. Completeness: surveying six data elements in one year dataset from all sample sites. Accuracy: surveying a random sample sites in three months to assess variation of three steps in data collection and reporting	Extracted one year dataset for surveying data completeness of six data elements. Randomization sampling. Paralleled collection of raw data by on-site audit of the original data. Reconstructed an objective, quality-assured “gold standard” report dataset. All clinical sites were surveyed for data completeness, 99 sites were sampled for data accuracy	Descriptive statistics, by using charts, average magnitude of deviation from expected, and data concordance analysis between reported data and reconstructed dataset	Large sample size, randomized sampling technique, the use of an objective, quality-assured “gold standard” report generated by on-site audit of the original data to evaluate the accuracy of data elements reported in the PHIS. Set up thresholds of accuracy and errors	Sources of data were not verified
Matheson *et al.* 2012 [71] *	Missing data, invalid data, data cleaning, data management processes	Not conducted	N/A	N/A	N/A	Lack of specific metrics
ME DQA 2008 [34]	Accuracy, reliability, precision, completeness, timeliness, integrity, confidentiality	Comprehensive audit in quantitative and qualitative methods including in-depth verifications at the service delivery sites; and follow-up verifications at the next level	4 methods for selection of sites including purposive selection, restricted site design, stratified random sampling, random sampling; the time period corresponding to the most recent relevant reporting period for the IS. Five types of data verifications including description, documentation review, trace and verification (recount), cross-checks, spot-checks. Observation, interviews and conversations with key data quality officials were applied to collect data	Descriptive statistics on accuracy, availability, completeness, and timeliness of reported data, including results verification ratio of verification, percentage of each dimension, differences between cross-check	Two protocols, 6 phases, 17 steps for the audit; sample on a limited scale considering the resources available to conduct the audit and level of precision desired; 2–4 indicators “case by case” purposive selection; on-site audit visits by tracing and verifying results from source documents at each level of the PHIS	Confined to specific disease context and standard program-level output indicators
ME PRISM 2010 [40]	Relevance: comparing data collected against management information needs. Completeness: filling in all data elements in the form, the proportion of facilities reporting in an administrative area. Timeliness: submission of the reports by an accepted deadline. Accuracy: comparing data between facility records and reports, and between facility reports and administrative area databases	Quantitative method, Questionnaire survey including data completeness and transmission, data accuracy check, data processing and analysis, assess the respondent’s perceptions about the use of registers, data collection forms and information technology	Non-anonymous interviews with identified name and title, including asking, manual counting, observation and recording results or circling “yes or no”	Using a data entry and analysis tool (DEAT), described in quantitative terms rather than qualitative. Yes or No tick checklist	A diagnostic tool in forms measures strengths and weaknesses in three dimensions of data quality. Quantitative terms help set control limits and targets and monitor over time	Indicators are not all inclusive; tool should be adapted in a given context. Need pre-test and make adjustments
Pereira *et al.* 2012 [72]	Completeness and accuracy of data-fields and errors	Quantitative and qualitative methods: Use primary (multi-center randomized trial) and secondary (observational convenience sample) studies	Field visits of a sample of clinics within each PHU to assess barcode readability, method efficiency and data quality. 64 clinic staff representing 65% of all inventory staff members in 19 of the 21 participating PHUs completed a survey examining method perceptions	Descriptive statistics: a weighted analysis method, histograms, 95% confidence intervals, F-test, Bootstrap method, the two-proportion z-test, adjusted the p values using Benjamin–Hochberg’s method for controlling false discovery rates (FDR)	The first study of such in an immunization setting.	Lack of representativeness to multiple lot numbers. Inaccurate data entry was not examined. Observations were based on a convenience sample
Petter and Fruhling 2011 [62]	Checklist of system quality, information quality	Quantitative methods to use DeLone&McLean IS success model. Use a survey in structured questionnaire	Online survey, facsimile, and mail, using 7 Likert scale for all quantitative questions. A response rate of 42.7% with representative demographics	Summative score for each construct, and each hypothesis was tested using simple regression. Mean, standard deviation, the Spearman’s correlation coefficients for analysis	Demonstrates the need to consider the context of the medical information system when using frameworks to evaluate the system	Inability of assessing some correlational factors due to the small PHIS user system
Ronveaux *et al.* 2005 [60]	Consistency The ratio of verified indicators reported compared with written documentation at health facilities and districts	Quantitative methods, using standardized data quality audits (WHO DQAs) in 27 countries	Recounted data compared to reported data	Descriptive statistics	A quantitative indication of reporting consistency and quality, facilitate comparisons of results over time or place	Similar to WHO DQA
Saeed *et al.* 2013 [61]	Completeness, validity, data management Calculation of missing data and illegal values (out of a predetermined range), data management (data collection, entry, editing, analysis and feedback)	Quantitative and qualitative methods, including interview, consultation, and documentation review	10 key informants interview among the directors, managers and officers; 1 or 2 staff at national level interviewed; consultation with stakeholders, document review of each system strategic plan, guidelines, manuals, annual reports and data bases at national level	Predefined scoring criteria for attributes: poor, average, or good	Comparison of two PHIS	Purposive sampling
Savas *et al.* 2009 [47]	Sensitivity, specificity and the Kappa coefficient for inter-rater agreement	Quantitative methods: audit data set by cross-linkage techniques	Databases were deterministically cross linked using female sex and social security numbers. Deterministic and probabilistic linkage methods were also compared	Descriptive statistics	Combined electronic databases provide nearly complete ascertainment for specific dataset	Using data which were missing would affect the results by under-ascertainment
Van Hest *et al.* 2008 [46]	Accuracy and completeness of reported cases	Quantitative methods: audit data set by record-linkage and capture-recapture techniques	Use record linkage, false-positive records and correction, and capture-recapture analysis through 3 data sources by a core set of identifiers	Descriptive statistics: number, proportion and distribution of cases, 95% ACI (Approximate confidence interval), Zelterman’s truncated model	Record-linkage of TB data sources and cross-validation with additional TB related datasets improves data accuracy as well as completeness of case ascertainment	Imperfect record-linkage and false-positive records, violation of the underlying capture–recapture assumptions
Venkatarao *et al.* 2012 [22]	Timeliness: Percentage of the reports received on time every week; Completeness: percentage of the reporting units sending reports every week	Quantitative methods: Use field survey (questionnaire) with a 4-stage sampling method	2 study instruments: the first focused on the components of disease surveillance; the second assessed the ability of the study subject in identifying cases through a syndromic approach	Descriptive statistics analysis	Two instruments including surveying users and dataset	Not able to assess the quality of data source such as accuracy
WHO DQA 2003 [42]	Completeness of reporting, report availability, timeliness of reporting, verification factor	Quantitative methods to audit selected indicators in the dataset. Multi-stage sampling from stratified sample representing the country’s PHIS	Recounted data compared to reported data	Descriptive statistics	A systematic methodology to describe data quality in the collection, transmission and use of information, and to provide recommendations to address them	Sample size and the precision dictated by logistical and financial considerations
WHO DQRC 2013 [44]	Completeness of reporting; internal consistency of reported data; external consistency of population data; external consistency of coverage rates	Quantitative method to conduct a desk review of available data and a data verification component at national level and sub-national level	An accompanying Excel-based data quality assessment tool	Simple descriptive statistics: percentage, standard deviation	Easy to calculate	Needs WHO DQA to complement assessment of the quality of data source
WHO HMN 2008 [45]	Data-collection method, timeliness, periodicity, consistency, representativeness, disaggregation, confidentiality, data security, and data accessibility.	Quantitative and qualitative methods to use 63 out of 197 questions among around 100 major stakeholders	Use consensus development method by group discussions, self-assessment approach, individual (less than 14) or group scoring to yield a percentage rating for each category	An overall score for each question, quartiles for the overall report.	Expert panel discussion, operational indicators with quality assessment criteria.	Sample size was dictated by logistical and financial considerations

**Table A2:** Characteristics of the methods for assessment of data use reported in the 10 publications included in the review.

Authors Year	Attributes Major measures	Study design	Data collection methods	Data analysis methods	Contribution	Limitations
Freestone *et al.* 2012 [52]	Trends in use Actioned requests from researchers in a set period of time	Analysis of actioned requests from researchers in a period of time	Abstracted data from the database for the study period	Trend analysis of proportion of requests	Quantifiable measures	Limit attributes
Hahn *et al.* 2013 [50]	Use of data The usage of aggregated data for monitoring, information processing, finance and accounting, and long-term business decisions	Qualitative methods: structured interviews with purposive sample of 44 staff and in-depth interviews with 15 key informants	Structured survey and key informant interview to assess five structured statements. Five-point scales were used for each statement	Responses were processed manually, classified and grouped by facility and staff class	Identified indicators of use of data	Lack of quantifiable results for assessment of data use
Iguiñiz-Romero and Palomino 2012 [70]	Data use Data dissemination: identify whether data used for decision making, the availability of feedback mechanisms	Qualitative exploratory study including interview and review of documentations	Open-ended, semi-structured questionnaire interviews with 15 key decision-makers. Review national documents and academic publications	Interview data recorded, transcribed, organized thematically and chronologically. The respondents were identified by positions but not named	Most respondents held key positions and a long period of the reviewed publications	Purposive sample lack of representativeness
Matheson *et al.* 2012 [71]	Clinical use of data: the number of summaries produced. Use of data for local activities to improve care. Data entry: the number of active sites. Report use: the percentage of active sites using prebuilt queries to produce data for each type of report in a given month over time	Qualitative and quantitative methods: key informant interview, documentation review, database query.	Personal interviews by phone and through internet telephony; follow up in person or by email; running SQL queries against the central database. External events were identified by reviewing news reports and through personal knowledge of the authors	Descriptive statistics using charts on number of clinics using the system in a given month, percentage of active clinics	Multiple methods	Lack of verification of data source
ME PRISM 2010 [40]	Checklist of use of information Report production, display of information, discussion and decisions about use of information, promotion and use of information at each level	Quantitative method to complete a predesigned checklist diagnostic tool	Checklist and non-anonymous interviewing staff, asking, manual counting, observation and recording results or circling “yes or no”	Two Likert score and descriptive statistics	Quantitative terms help set control limits and targets and monitor over time	
Petter and Fruhling 2011 [62]	System use, intention to use, user satisfaction	Quantitative methods to use DeLone & McLean IS success model. Survey respondents with a response rate of 42.7% and with representative demographics	Use an online survey in structured questionnaire with 7 Likert scale for all quantitative questions, in addition to facsimile and mail	Summative score for each construct, and each hypothesis was tested using simple regression, in addition to mean, standard deviation, the Spearman’s correlation coefficients	Use is dictated by factors outside of the control of the user, and it is not a reasonable measure of IS success. The quality does not affect the depth of use	Lack of objective assessments
Qazi and Al 2011 [27]	Use of data Non-use, misuse, disuse of data	Descriptive qualitative interviews	In-depth, face to face and semi structured interviews with an interview guide, 26 managers (all men, ages ranging from 26 to 49 years; selected from federal level (2), provincial (4) and seven selected districts (20) from all four provinces)	Data transcription, analysis based on categorization of verbatim notes into themes and a general description of the experience that emerged out of statements	A qualitative study allows getting close to the people and situations being studied, identified a number of hurdles to use of data	Convenience sample only one type of stakeholders has been covered.
Saeed *et al.* 2013	Usefulness of the system Data linked to action, feedback at lower level, data used for planning, detect outbreaks, data used for the development and conduct of studies	Quantitative and qualitative methods, including interview, consultation, and documentation review	10 key informants interview; consultation with stakeholders, document review of each system	Predefined scoring criteria for attributes: poor, average, or good	Mixed methods	Purposive sampling
WHO HMN 2008 [45]	Information dissemination and use, demand and analysis, policy and advocacy, planning and priority-setting, resource allocation, implementation and action	Mixed methods: quantitative and qualitative. Use 10 out of 197 questions among stakeholders at national and subnational levels	Use group discussions (100 major stakeholders), self-assessment approach, individual (less than 14) or group scoring to yield a percentage rating for each category	An overall score for each question, quartiles for the overall report	Expert panel discussion, operational indicators with quality assessment criteria	Lack of field verification of data use
Wilkinson and McCarthy	Extent of data recognition and use, strategies and routines, specific uses, dissemination	Quantitative and qualitative methods to use standardized semi-structured questionnaire telephone interviews of key informants from the management teams of the system	Telephone structured questionnaire interviews of 68 key informants from the 29 out of 34 management teams of the networks. Response options for most of the questionnaire items were yes/no or five or seven point Likert and semantic differential response scales	Quantitative and qualitative analysis of survey results. Qualitative data transcribed, ordered by question number, and common themes, then content analyzed to indicate frequencies and percentages. Correlational analyses used Pearson’s r for parametric data and Spearman’s Rho for non-parametric data	Quantification of qualitative data	Statistical analysis is limited by the size of the sample as there were only 29 networks and 68 individual participants, statistical power to detect an effect is weak, and general trends are mainly reported.

**Table A3:** Characteristics of the methods for assessment of data collection process reported in the 16 publications included in the review.

Authors Year	Attributes Major measures	Study design	Data collection methods	Data analysis methods	Contribution	Limitations
Ancker *et al.* 2011 [59]	Group discussion about root causes of poor data quality and strategies for solving the problems	Qualitative method by focus group discussion	Held a series of weekly team meetings over about 4 months with key informants involved in the data collection	Theme grouping to each data quality issue	Initiated by and related to identified poor data quality issues	Implicitly focused. Only analyzed causes not assessed the magnitude
Bosch-Capblanch *et al.* 2009 [58]	Quality scores Recording and reporting of data, keeping of vaccine ledgers and information system design	Quantitative method by user’s survey based on WHO DQA. A multistage weighted representative sampling procedure	Questionnaire based on a series of 19 questions and observations undertaken at each level (national, district and health units)	Each question 1 point. Average score, summary score, medians, inter-quartile ranges, confidence intervals, P value, bubble scatter chart, Rho value	Combined with data quality	Implicitly focused, the number of questions surveyed was less than that of the WHO DQA
CIHI 2009 [30]	Metadata documentation Data holding description, methodology, data collection and capture, data processing, data analysis and dissemination, data storage, and documentation.	Quantitative method by surveying users	Questionnaire	Undefined	7 categories, with subcategories and definition and/or example	Implicitly focused
Corriols *et al.* 2008 [55]	Identification of underreporting reasons by reviewing information flow chart and non-reporting in physicians	Qualitative method to review documentations	Review the national reports on the system related to deficiency in the information flow chart and non-reporting in physicians	Undefined	Initiated by identified data quality issues	Implicitly focused
Dai *et al.* 2011 [69]	Data collection, data quality management, statistical analysis and data dissemination	Qualitative method, review documentations	Document review	Theme grouping	Desk review	Implicitly focused
Forster *et al.* 2008	Routine data collection, training and data quality control	Quantitative method by online survey	Questionnaire	Descriptive statistics.	Examine associations between site characteristics and data quality	Implicitly focused. Convenience sample
Freestone *et al.* 2012 [52]	Data collection and recording processes	Qualitative method to review current processes about identification, code, geocode of address or location data. Staff consulted to establish and observe coder activities and entry processes	Review the processes; consultation with staff; observation of coder activities and entry processes to identify any potential cause of errors which then grouped thematically	Thematically grouping data	Identify each of the key elements of the geocoding process are factors that impact on geocoding quality	Differences in software and system settings need to be aware of.
Hahn *et al.* 2013 [50]	Data flow The generation and transmission of health information	Qualitative method to use workplace walkthroughs on 5 subsequent working days at each site	Informal observations of the generation and transmission of health information of all kinds for the selection of data flows	Undefined	Observation of walkthroughs	Undefined indicators
Iguiñiz-Romero and Palomino 2012 [70]	Data flow or data collection process: data collectors, frequencies, data flow, data processing and sharing,	Qualitative exploratory study including interview and review documentations	Open-ended, semi-structured questionnaire interviews with 15 key decision-makers. Review national documents and academic publications	Data recorded, transcribed, organized thematically and chronologically	Most respondents held key positions and a long period of reviewed publications	Purposive sample
Lin *et al.* 2012 [65]	Data collection and reporting	Qualitative methods based on CDC’s Guidelines,	Review guidelines and protocols using a detailed checklist; direct observation; focus group discussions and semi-structured interviews	Theme grouping	Field visits or observations of data collection to identify impact on the data quality	Undefined indicators
ME DQA 2008 [34]	Five functional areas: M&E structures, functions and capabilities, indicator definitions and reporting guidelines, data collection and reporting forms and tools, data management processes, and links with national reporting system	Quantitative and qualitative methods by 13 system assessment summary questions based on 39 questions from five functional areas. Score the system combined with a comprehensive audit of data quality	Off-site desk review of documentation provided by the program/project; on-site follow-up assessments at each level of the IS, including observation, interviews, and consultations with key informants	Using summary statistics based on judgment of the audit team. Three-point Likert scale to each response. Average scores for per site between 0 and 3 continuous scale	DQA protocol and system assessment protocol	Implicitly focused. The scores should be interpreted within the context of the interviews, documentation reviews, data verifications and observations made during the assessment.
ME PRISM 2010 [40]	ProcessesData collection, transmission, processing, analysis, display, quality checking, feedback	Quantitative method by questionnaire survey including data transmission, quality check, processing and analysis and assessing the respondent’s perceptions about the use of registers, data collection forms and information technology	Non-anonymous interviewing staff with identified name and title, including asking, observation and circling “yes or no”	Using a data entry and analysis tool (DEAT), described in quantitative terms rather than qualitative. Yes or No tick checklist	A diagnostic tool. Quantitative terms help set control limits and targets and monitor over time	Indicators are not all inclusive; tool should be adapted and pre-test and make adjustments
Ronveaux *et al.* 2005 [60]	Quality index (QI) Recording practices, storing/reporting practices, monitoring and evaluation, denominators used at district and national levels, and system design at national level	Quantitative and qualitative methods by external on-site evaluation after a multi-stage sampling based on WHO DQA.	Questionnaires and observations. Survey at national level (53 questions), district level (38 questions) and health-unit level (31 questions). Observations to workers at the health-unit level. They were asked to complete 20 hypothetical practices.	Descriptive statistics (aggregated scores, mean scores): 1 point each question or task observed. Correlational analyses by zero-order Pearson correlation coefficients		Implicitly focused. The chosen sample size and the precision of the results were dictated by logistical and financial considerations
Venkatarao *et al.* 2012 [22]	Accuracy of case detection, data recording, data compilation, data transmission	Quantitative method by using a 4-stage sampling method to conduct field survey (questionnaire) during May-June 2005 among 178 subjects	Questionnaires of 2 study instruments: the first focused on the components of disease surveillance; the second assessed the ability of the study subject in identifying cases through a syndromic approach	Descriptive statistics analysis	Assessment from user’s viewpoint.	Implicitly focused. Lack of field verification of data collection process
WHO DQA 2003 [42]	Quality questions checklist, quality index Five components: recording practices, storing/reporting practices, monitoring and evaluation, denominators, system design (the receipt, processing, storage and tabulation of the reported data)	Quantitative and qualitative method using questionnaire checklists for each level (three levels: national, district, health unit level) of the system including 45, 38, 31 questions respectively	Questionnaires and discussions. Observations by walking around the health unit for field observation to validate the reported values	Percentage of the items answered yes. The target is 100% for each component	Describe the quality of data collection and transmission	Implicitly focused. The chosen sample size was dictated by logistical and financial considerations
WHO HMN 2008 [45]	Data management or metadata A written set of procedures for data management including data collection, storage, cleaning, quality control, analysis and presentation for users, an integrated data warehouse, a metadata dictionary, unique identifier codes available	Mixed methods: quantitative and qualitative. Use 5 out of 197 questions, at various national and subnational levels	Use group discussions around 100 major stakeholders, self-assessment approach, individual (less than 14) or group scoring to yield a percentage rating for each category	An overall score for each question, quartiles for the overall report	Expert panel discussion, operational indicators with quality assessment criteria	Lack of field verification of data collection process
